# The roles of hepatokine and osteokine in liver-bone crosstalk: Advance in basic and clinical aspects

**DOI:** 10.3389/fendo.2023.1149233

**Published:** 2023-04-06

**Authors:** Zhanghao Li, Xiaoxin Wen, Nanxi Li, Chuanxin Zhong, Li Chen, Feng Zhang, Ge Zhang, Aiping Lyu, Jin Liu

**Affiliations:** ^1^ Law Sau Fai Institute for Advancing Translational Medicine in Bone and Joint Diseases (TMBJ), School of Chinese Medicine, Hong Kong Baptist University (HKBU), Hong Kong, Hong Kong SAR, China; ^2^ Department of Anatomy, Jinzhou Medical University, Jinzhou, China; ^3^ Jiangsu Key Laboratory for Pharmacology and Safety Evaluation of Chinese Materia Medica, Nanjing University of Chinese Medicine, Nanjing, China; ^4^ Guangdong-Hong Kong-Macau Joint Lab on Chinese Medicine and Immune Disease Research, Guangzhou, China

**Keywords:** liver, bone, hepatokine, osteokine, endocrine

## Abstract

Both the liver and bone are important secretory organs in the endocrine system. By secreting organ factors (hepatokines), the liver regulates the activity of other organs. Similarly, bone-derived factors, osteokines, are created during bone metabolism and act in an endocrine manner. Generally, the dysregulation of hepatokines is frequently accompanied by changes in bone mass, and osteokines can also disrupt liver metabolism. The crosstalk between the liver and bone, particularly the function and mechanism of hepatokines and osteokines, has increasingly gained notoriety as a topic of interest in recent years. Here, based on preclinical and clinical evidence, we summarize the potential roles of hepatokines and osteokines in liver-bone interaction, discuss the current shortcomings and contradictions, and make recommendations for future research.

## 1 Introduction

Multiple factors could lead to liver diseases with visible extrahepatic complications, influencing disease progression and therapy efficacy. Reduced bone mineral density (BMD) caused by both decreased physiological bone turnover and limited osteosynthesis processes is one of the most common extrahepatic complications found in adults suffering from end-stage liver disease (1). In addition, most patients suffering from liver disease also have multiple risk factors for osteodystrophy, including protein-calorie malnutrition and vitamin D deficiency (2). Hepatic osteodystrophy (HOD), a term for bone loss and fractures caused by liver disease (3), occurs in up to 50% of patients undergoing chronic liver disease (CLD) (4). Typical liver-derived hormone fibroblast growth factor 21 (FGF21) could inhibit osteoblast activity and increase bone resorption (5). In terms of hepcidin, the other hepatokine, its deficiency led to the reduction of bone formation. Thus, it is undoubted that abnormal liver disease and bone metabolism are closely related.

Apart from the key component of motor system, bone has been recognized as an endocrine organ that regulates energy metabolism (6). For instance, osteocytes and osteoblasts secreted FGF23 is positively correlated with liver fat content (7). Furthermore, osteoblasts-expressed osteocalcin (OCN) has also been reported to increase insulin secretion and sensitivity (8). The secreted bone factor LCN2 conditional knockout mice in osteoblasts showed increased blood glucose levels and body fat (9). Other bone-derived factors, such as calcitonin and sclerostin (SOST), are also closely linked to liver metabolism. Calcitonin knockout mice were protected against high-fat diet (HFD)-induced obesity. After receiving SOST-neutralizing antibody, mice were also resistant to diet-induced insulin resistance, indicating the possible role of osteokines in regulating liver metabolism. However, liver abnormalities are frequently underestimated as a side effect of medication for treating bone diseases rather than direct harm caused by the abnormal bone metabolism characterized by excessive bone resorption and/or constrained bone formation (10). Emerging evidence has advocated that liver and bone are mutually regulated through liver-bone crosstalk, a constant interorgan communications mediated by the hepatic and osteal endocrine factors. The physiological liver-bone crosstalk is important for liver and bone health while the pathological liver-bone crosstalk could substantially contribute to the development of bone-related comorbidities in liver diseases, i.e., HOD. In this review, we focused on research progress regarding the roles of hepatokine and osteokine in liver-bone crosstalk and their contributions to liver disease associated with abnormal bone metabolism or abnormal liver glucose and lipid metabolism of bone disease, respectively. We thoroughly analyzed direct evidence from basic studies and indirect evidence from clinical studies. Besides, some seemingly contradictory findings in basic and clinical studies were also summarized and discussed.

## 2 Hepatokines: From liver to bone

### 2.1 Fibroblast growth factor 21

Fibroblast growth factor 21 (FGF21) is a member of the FGF subfamily produced primarily in the liver. Its major function is to regulate the energy metabolism of carbohydrates and lipids in the body through endocrine and other mechanisms (5, 11–13). The deficiency of FGF21 facilitates the development of steatosis, inflammation, hepatocyte damage, and fibrosis in the liver (14, 15). Supplementation of FGF21 or its analogists could be conductive to alleviating the development of nonalcoholic steatohepatitis (NASH) (16). However, studies have shown that FGF21 makes deleterious effects on bone mass (17, 18). Compared with the wild-type controls, the transgenic mice with overexpressed FGF21 had a striking decrease in trabecular bone mass. Meanwhile, FGF21-knockout mice developed a high-bone-mass phenotype (19). Reduction of FGF21 levels in serum and liver was capable of preventing osteoclastogenesis and bone loss *via* tail vein injection of agomir-miR-100-5p, which could combine with 3’ UTR of FGF21 (17). Due to the obviously negative correlation between FGF21 and bone mass, the mechanism of FGF21 in regulating bone homeostasis has aroused researchers’ interest. Wei et al. confirmed that overexpressed circulating FGF21 stimulated lipogenesis of bone marrow precursors, inhibited osteoblast activity, and increased osteoclast activity by enhancing peroxisome proliferator-activated receptor γ (PPARγ) activity in bone marrow mesenchymal stem cells (19). Wang et al. identified that the pro-osteoclastogenic activity of FGF21 was related to insulin-like growth factor binding protein 1 (IGFBP1). In the transgenic mice with overexpression of FGF21, IGFBP1 secretion was indirectly induced, and IGFBP1 bound to osteoclast precursor integrin β1, enhancing the receptor activator of nuclear factor κB ligand (RANKL)-stimulated extracellular regulated protein kinases phosphorylation and NASH nuclear factor of activated T cells 1 activation, ultimately promoting osteoclast differentiation and resulting in decreased BMD (18).

However, different from the clear and overwhelming evidence in animal research, there is little clinical research, and the research results remain controversial. Overweight subjects with type 2 diabetes experienced increased bone resorption marker C-telopeptide of type I collagen after administration of a long-acting FGF21 analog PF-05231023, as well as the lower circulating levels of IGF1 and various markers of bone formation, including OCN, P1NP, and bone-specific alkaline phosphatase (46). Elevated FGF21 levels in individuals with HIV-1 infection are strongly associated with increased bone resorption, making it a potential biomarker for disrupted bone homeostasis and indicators of metabolic derangement (47). However, in younger adults between 22-39 years old and older adults between 60-71 years old, the relationship between FGF21 and IGFBP1 appears to differ from the previous studies (18). According to Lee et al., there was a negative relationship between BMD at the spine and the circulating FGF21, while no association was found between BMD at the hip or spine and IGFBP1 (48). In middle-aged and elderly European men (between the ages of 40 and 79 years), there was a negative correlation between serum IGFBP1 concentrations and calcaneal BMD (49). Moreover, it remains to validate whether the FGF21-IGFBP1-RANKL pathway is involved in age-related bone loss in human. In contrast, another study revealed a significantly positive correlation between plasma FGF21 levels and total BMD in healthy women (50). In Chinese Han postmenopausal women, Hu et al. discovered that serum FGF21 concentrations were positively associated with lumbar spine BMD, but not with bone turnover markers or with fragility fracture (51). In a clinical trial of obese children and adolescents, there was no correlation between FGF21 levels and antero-posterior vertebral L2-4 BMD z-score values (52). As a result, it is unclear whether the inner linkage between FGF21 and IGFBP1 still exists in the clinical aspect. Different populations, such as age groups, should be included and considered as interfering factors. Moreover, further clinical research is needed to fully understand the role of FGF21 and bone loss in physiological and pathological conditions, particularly whether the association is limited to a specific pathological condition, such as HOD.

### 2.2 Bone morphogenetic protein 9

Even though the precise distribution varies slightly between species (53), in general, bone morphogenetic protein 9 (BMP9) is considered to be mainly expressed in the liver of adult individuals as a circulating factor produced by hepatic stellate cells (54). In managing liver sinusoidal endothelial cells fenestration and guarding against perivascular hepatic fibrosis, it is a crucial paracrine regulator of liver homeostasis (55, 56). In nonalcoholic fatty liver disease (NAFLD) patients and animal models, BMP9 levels were lower in the liver and serum. BMP9-knockout mice also exhibit hepatosteatosis because of down-regulated PPARα expression and reduced fatty acid oxidation (20). In addition, BMP9-knockout mice featured alveolar bone with reduced volume, decreased mineral density, and trabecular thickness (57). As the strong inducer of osteocyte differentiation in the BMP family, BMP9 is a crucial regulator of skeletal homeostasis (54, 58). In osteoporotic rats with femora fractures, global overexpression of BMP9 by adenovirus (Ad) mediated callus formation and increased bone mass and strength to great extent *via* promoting osteoblastic differentiation (59). Similarly, Zhou et al. proved that overexpression of BMP9 led to not only elevated BMP9 levels in the liver and serum but also suppressed bone resorption activity, improved cortical and trabecular volumetric BMD, and ameliorated bone strength in an ovariectomy mouse model (21, 60). BMP9 increased bone mass in aged mice by preventing osteoblast senescence and stimulating osteoblast differentiation, improved bone biomechanical properties, and ameliorated the bone microenvironment (61). Aside from directly influencing bone resorption and bone formation, BMP9 upregulated the endogenous expression of RUNX3 in mesenchymal stem cells (62), which are undifferentiated stem cells with the potential to differentiate into multiple lineages, including osteoblasts (63). The *in vivo* liver-specific BMP9 knockout model should be established to investigate the role of BMP9 in liver-bone crosstalk. Besides, more clinical research should be performed to determine whether BMP9 derived from liver non-parenchymal cells is linked to bone disease. Considering the pro-anabolic and anti-resorptive effect of BMP9 on bone, manipulating BMP9 could be a potential therapeutic strategy for bone loss associated with liver disease.

### 2.3 Vitamin D

Vitamin D (VitD) plays an active role in immune function, protein synthesis, cardiovascular function, and musculoskeletal regulation. In the healthy liver, VitD is hydroxylated by VitD 25-hydroxylase (CYP2R1) and sterol 27-hydroxylase (CYP27A1). The expression of both enzymes decreased in fibrotic and cirrhotic livers (64, 65). VitD binding protein, which is synthesized by the liver and acts in binding and transporting VitD, is shown to be low-expressed in sepsis-induced liver injury (66). Thus, the metabolism and transportation of VitD are impaired in advanced liver disease (67, 68). VitD is also key to bone mineral homeostasis. When VitD levels decrease below normal limits, parathyroid hormone increases bone resorption to satisfy the body’s demands for calcium, increasing bone turnover with an added risk of bone fracture (22). An observational study verified that VitD deficiency was closely related to HOD, which is a metabolic bone disease often associated with chronic liver disease and is marked by the bone loss (69). Compared to patients with Child-Pugh grades A and B cirrhosis, lumbar BMD and VitD active metabolites were extremely lower, whereas the bone resorption marker β-CTX was higher, in patients with grade C cirrhosis (70), suggesting that cirrhosis is a risk factor for osteoporosis and VitD level could be an important marker for evaluating HOD. At present, there is no direct study to demonstrate that liver regulation VitD in chronic liver disease is involved in hepatic bone disease, and further confirmation is necessary. Calcium and/or VitD supplementation may be of importance in reducing HOD, but its safety and efficacy must also be taken into consideration to prevent the hypercalcemia, hypercalciuria, and hyperphosphatemia that can be brought on by prolonged vitamin D supplementation.

### 2.4 Fetuins

Both fetuins-A and fetuins-B are important hepatokines in human metabolism regulation (71). Fetuins-A, also known as Alpha-2-HS-glycoprotein, is mainly expressed, and secreted by the liver and adipose tissue (72). The upward fetuin-A serum level is correlated with high liver fat content (25, 26). As a mineral carrier protein and the inhibitor of pathological soft tissue calcification, it is also enriched in bone (27). Heterotopic ossification (HO) is the abnormal formation of bone in extraskeletal sites. Therapy with recombinant fetuin-A prevents injury-induced and BMP4-dependent HO and associated bone loss through increasing the expression of programmed cell death protein 1 reducing macrophage infiltration and inhibiting hyperinflammation (72). Several studies have suggested that fetuin-A was intimately associated with BMD (27, 73, 74). A single dose of bovine fetuin-A could reduce the visible osteolytic lesions and eroded bone surface in mice subjected to particle-induced osteolysis (27). However, in a large sample of community-dwelling older adults, fetuin-A was positively associated with areal BMD in a small degree, and there was no evidence of an association between fetuin-A and the risk of clinical fracture (75). The liver-specific fetuins-A overexpression mice may be an interesting model in further investigation. Like fetuin-A, fetuin-B is also a liver-derived plasma protein. It is increased in patients with type 2 diabetes and impairs insulin sensitivity in myotubes and hepatocytes (76, 77). A 4-year prospective study in China showed serum level of fetuin-B is associated with osteoporosis (78). Nevertheless, research on the role of fetuin-B in bone metabolism remains limited, both *in vivo* and *in vitro*. Further research is required to determine the relationship between fetuin-B and bone mass.

### 2.5 Hepcidin

Hepcidin, a key regulator of iron metabolism, is primarily synthesized in hepatocytes and has emerged as a new marker of fibrosis and cirrhosis (79). Patients with CLD usually had lower serum levels of hepcidin (80). Overexpression of hepcidin increased adiponectin expression in hepatocytes and hepcidin treatment inhibited hepatic stellate cells activation, thus alleviating liver fibrosis (28). Interestingly, in a recent study, the relationship between hepcidin and bone loss has been revealed. Hepcidin^-/-^ mice as iron overload models, showed the phenotype of reduced bone formation and enhanced bone resorption, which could be caused by the increased reactive oxygen species and resultant sclerostin (SOST) and RANKL/OPG expression alteration (81). Guo et al. created TgHamp1-Alb mice that specifically express hepcidin in liver hepatocytes by hybridization of LSL-Hamp1 (TgHamp1) mice with albumin (Alb) promoter-driven Cre (Alb-Cre). At 3 months old, TgHamp1-Alb mice displayed the reduced trabecular volume/total volume, trabecular number, and trabecular thickness (29). Min et al. pointed out that the increased hepcidin levels were closely related to reduced 25‐hydroxyvitamin D in chronic kidney disease (CKD), further causing CKD-related bone fracture (82). Clinical research on hepcidin in bone loss is still lacking. The development of *in vivo* models for hepatocytes hepcidin knockout or overexpression is fundamental to determining whether liver-derived hepcidin affects bone loss.

### 2.6 Lecithin-cholesterol acyltransferase

As a liver-derived enzyme (83), the lecithin cholesterol acyltransferase gene (LCAT) plays an important role in lipoprotein metabolism (84). Low LCAT activity may be the cause of the lipoprotein changes in parenchymal liver disease (85, 86). The loss of LCAT in progressive liver injury caused worse liver fibrosis and HOD, and markedly exacerbated the bone loss phenotype. Tail vein rAAV8-LCAT or recombinant LCAT (rLCAT) injection significantly increased bone mass and inhibited osteoclastogenesis in HOD mice (83, 87). At present, the mechanistic understanding of LCAT on bone metabolism are limited to the regulation of cholesterol metabolism in osteoblasts and osteoclasts. rLCAT dramatically lowered the intracellular cholesterol in primary osteoclasts isolated from mice and disrupted estrogen-related receptor alpha transcription, thereby inhibiting osteoclast differentiation. Cholesterol treatment prevented osteoblast differentiation by decreasing the mRNA levels of osteoblast marker genes, but rLCAT treatment could restore osteoblast differentiation and reduce intracellular cholesterol. However, the target of LCAT needs to be further defined (87). Moreover, there is still a lack of clinical evidence linking LCAT to bone loss. In this case, further research is needed to ascertain the precise mechanism by which LCAT maintains bone homeostasis, whether and to what extent LCAT-mediated bone metabolism depends on cholesterol metabolism, and whether LCAT having the potential to serve as a marker of bone loss associated with liver disease. The findings of hepatokines are summarised in **Table 1**.

**Table 1:** Hepatokines involved in bone regulation.

Hepatokines	The effect on liver	The effect on bone	Reference
FGF21	Alleviate the development of NASH	Increase osteoclastogenesis and bone loss	(18)
BMP9	Inhibit hepatosteatosis	Promote bone formation and suppress bone resorption	(20, 21)
VitD	Inhibit insulin resistance, alleviate severity of steatosis, necroinflammation and fibrosis in NAFLD	Stimulate osteoblast maturation, mineral deposition, and osteoclastogenesis	(22–24)
Fetuins	Increase liver fat content	Reduce the visible osteolytic lesions in particle-induced osteolysis and prevent injury-induced and BMP4-dependent HO and associated bone loss	(25–27)
Hamp	Alleviate liver fibrosis	Enhance osteoclastogenesis	(28, 29)
LCAT	Promote lipoprotein metabolism	Inhibit osteoclastogenesis and restore osteoblast differentiation in HOD	(30, 31)

## 3 Osteokines: From bone to liver

### 3.1 Fibroblast growth factor 23

Fibroblast growth factor 23 (FGF23) is a bone-derived hormone secreted by osteocytes and osteoblasts binding to FGF receptor-Klotho complexes (88). It interferes with osteoblast differentiation and matrix mineralization and increases renal phosphate excretion (32, 89). As an important marker of CKD, rising FGF23 levels indicated appropriate compensation to maintain a neutral phosphorus balance in renal dysfunction (90). High levels of serum FGF23 are associated with hypophosphataemia-related rickets (91), as well as the autosomal dominant hypophosphataemic rickets (ADHR), which is characterized by mutations of two FGF23 cleavage sites Arg179 and Ser180 and is frequently accompanied by markedly elevated serum ALP, which may reflect underlying liver abnormalities (92). Massive elevations in circulating FGF23 contribute to the elevation of liver inflammation, whereas the normal mouse liver itself possesses very low levels of FGF23 mRNA and protein (33, 93). FGF23 could stimulate calcineurin signaling by activating FGF receptor isoform 4 in cultured hepatocytes, which increased the expression and secretion of inflammatory cytokines (94). Besides, it also directly regulated liver fetuin-A expression. When FGF23 increased up to 400-600 pg/mL, fetuin-A increased progressively and declined at higher FGF23 concentrations (95), which may act in a bidirectional manner to control fetuin-A’s involvement in hepatic glucose homeostasis. In Chinese diabetes mellitus type 2 patients, both high FGF23 and low VitD levels had an independent relationship with NAFLD (96). Furthermore, a community-based cohort also revealed that FGF23 in serum was positively related to MAFLD and liver fat content (7). Although FGF23 is involved in the regulation of bone-liver axis, it has received less attention in liver diseases than in kidney disease. In addition, there are not enough reliable *in vivo* experimental and clinical data to consolidate the role of FGF23 in liver pathology. Thus, it would be valuable to exam the glucose lipid metabolism and inflammatory microenvironment in liver of bone-specific FGF23 knockout mice and validate the association between FGF23 and various liver diseases in clinical study.

### 3.2 Osteocalcin

Osteocalcin, also known as bone γ-carboxyglutamate protein (Bglap), is the most abundant osteoblast-specific non-collagenous protein that is expressed by osteoblasts (97). As a determinant of bone formation, the osteocalcin-deficient mouse is characterized by increased cortical thickness and trabecular bone mass (98). Besides its known role in bone health, osteocalcin may influence glucose homeostasis (99). Several studies have demonstrated a protective effect of osteocalcin against NAFLD (35, 36). In wild-type mice fed a western diet, osteocalcin increased the secretion and sensitivity of insulin (8, 100), reduced hepatic sterol regulatory element-binding protein-1 (101), triglyceride accumulation, malondialdehyde as well as the ratio of oxidized to reduced glutathione (102). Osteocalcin robustly reduced the expression of proinflammatory and profibrotic genes in liver of Ldlr^-/-^ mice fed with a high-fat, high-cholesterol diet for 12 weeks to induce metabolic syndrome and NASH (103). In old laying hens with fatty liver hemorrhagic syndrome, osteocalcin restricted the metabolic disorder, oxidative stress, and related pathological damage (104). In addition, a putative receptor of osteocalcin, GPRC6A, was found in the liver. With the construction of liver-specific GPRC6A knockout mice, Zhang et al. demonstrated that intraperitoneal injection of osteocalcin significantly protected wild-type mice from obesity and NAFLD, but not liver-specific GPRC6A knockout mice, suggesting that GPRC6A mediated the ability of osteocalcin to inhibit lipid synthesis and promote lipolysis (35). A murine circulating pentadecapeptide derived from pre-osteocalcin, binding to GPRC6A, could also alleviate the symptoms of NAFLD by inhibiting lipid absorption and insulin resistance (36). Consistent with the results from preclinical studies, osteocalcin was also found to negatively correlate with NAFLD in several clinical studies. In men with NAFLD or postmenopausal women, the large N-mid fragment of osteocalcin was negatively related to the probable presence of significant fibrosis or probable NASH (105). In a South Korean study involving 7,067 women, osteocalcin was also linked to insulin resistance. The serum osteocalcin level was found to be an independent risk factor for NAFLD (106). Further prospective clinical and in-depth animal studies are required to understand the relationship and underlying mechanisms of osteocalcin and liver disease progression. Moreover, the development of osteocalcin-specific receptor-targeted binding peptides in the liver will also be conducive to the treatment of glucose and lipid metabolism disorders.

### 3.3 Calcitonin

Calcitonin inhibits calcium efflux from bone rapidly lowers circulating calcium levels (107), and suppresses bone resorption through its corresponding receptor, the calcitonin receptor (CTR) (108). Calcitonin deficiency has been implicated in the pathogenesis of accelerated bone loss. Clinically, calcitonin has been adopted for conditions of accelerated bone turnover for several years, including Paget’s disease and osteoporosis (109). Compared with wild-type old age mice, calcitonin knockout mice, lost weight and had significantly lower levels of liver fat, adipocyte droplets, and lipids after HFD induction, and were protected against HFD-induced insulin resistance. The *in vitro* study verified that physiological concentrations of calcitonin promoted lipid accumulation, and suppressed adiponectin release in 3T3-L1 cells (110). However, in obese HFD-fed rats, although rat calcitonin (rCT) treatment made no effect, the combination of dual amylin and calcitonin receptor agonists (DACRAs) KBP-088, rat amylin (rAMY) significantly ameliorated rat glucose tolerance (111). The administration of DACRA combined with liraglutide for obese rats generated a significant effect on appetite suppression and body weight loss (112). The role of calcitonin in the liver and systemic glucose metabolism, as well as insulin resistance, appears to be conflicting. Moreover, it is still unknown whether calcitonin is used clinically to prevent bone calcium loss and whether it causes unexpected insulin resistance or improves insulin sensitivity. Therefore, more clinical data are required to observe the application of calcitonin. Furthermore, calcitonin and its splicing products could also be synthesized by hepatocytes, and the liver also possessed related receptors in non-parenchymal cells (113). Besides, the regulatory effect of calcitonin in liver on systemic metabolism is still insufficient.

### 3.4 Sclerostin

Sclerostin (SOST), which is mostly expressed in osteocytes, suppresses bone formation *via* the inhibition of the Wnt-low-density-lipoprotein receptor-related protein (LRP) 5/6 signaling pathway on osteoblasts (41). At present, the anti-SOST monoclonal antibody is used to treat severe osteoporosis (114–116). The bone volume in Sost^-/-^ mice experienced a marked increase, along with decreased accumulation of adipose tissue and increased insulin sensitivity. After administration of SOST-neutralizing antibody, mice were resistant to obesogenic diet-induced disturbances in metabolism. After treatment with recombinant sclerostin, Bmp4, Bmpr1a and Smad1/5/9 phosphorylation levels were increased in cultured primary adipocytes. Wnt3a treatment produced the opposite effect. Thus, sclerostin favors adipogenesis and adipose hypertrophy *via* the suppression of Wnt signaling (117). In a cross-sectional observational study, elevated SOST levels in alcoholics are linked to abnormal liver function, fat deposition, and elevated BMI (118). Serum SOST levels were also significantly higher in patients with cirrhosis (119), besides, it could reflect altered bone microarchitecture in those patients (120). However, another study showed that circulating SOST levels were significantly lower in NAFLD subjects compared with normal controls, which may mainly reflect reduced SOST secretion from bone tissues (121). Whether circulating SOST can be taken as a surrogate marker of bone metabolic status in patients with liver injury is uncertain, and the exact mechanism is still unknown. Furthermore, whether anti-SOST monoclonal antibodies can delay liver damage while treating severe osteoporosis is worth investigating.

### 3.5 Lipocalin 2

Lipocalin 2 (LCN2), which is also termed 24p3 or neutrophil gelatinase-associated lipocalin, was one of the adipokines (122). As a secreted bone factor, LCN2 positively affected osteogenesis *in vivo* and osteogenic differentiation of MC3T3-E1 (44). Mice who had LCN2 in their osteoblasts conditionally knocked out showed increased blood glucose levels and body fat. It was discovered that the appetite was directly suppressed by the melanocortin receptor 4 (MC4R) in the paraventricular and ventromedial neurons of the hypothalamus, which was bound by osteoblasts-derived LCN2. Additionally, wild-type mice receiving LCN2 continuously were featured with enhanced glucose metabolism and increased energy expenditure while reducing food intake, fat mass, and body weight (9). The expression of the chemokine receptor CXCR2 was also increased by lipocalin-2, thereby activating the mitogen-activated protein (MAP) kinase and extracellular regulated protein kinases 1/2 and producing proinflammatory chemokines, and aggravating steatohepatitis (45). LCN2 can also act as a key mediator of HSC activation in leptin-deficient obesity *via* α-SMA/MMP9/STAT3 signaling and accelerated NASH (123). In clinical research, higher blood level of LCN2 was linked to obesity, insulin resistance, and dyslipidemia in people with type 2 diabetes (124, 125). In patients with alcoholic hepatitis (AH), hepatic LCN2 expression and serum LCN2 levels both dramatically increased and were connected with disease severity and portal hypertension (45, 126, 127) LCN2 levels in the blood are a biomarker for metabolic diseases as a result and are expected to be a new therapeutic target to control appetite and obesity (8). Further investigation is needed in both experimental and clinical studies.

### 3.6 Transforming growth factor β

In healthy subjects, transforming growth factor β (TGF-β) is by far the most abundant cytokine in bone (42), which is secreted in its latent form by osteoblasts and osteoclasts. Upon secretion, the latent TGF-β is incorporated into the bone matrix (128). During bone resorption or fracture, osteoclasts activate TGF-β in their resorption lacuna *via* proteolytic and acidic hydrolysis (65), and it is also considered to be the major factor regulating liver carcinogenesis and accelerating liver fibrosis (43). In terms of activation of TGF-β, it promotes liver fibrosis by activating HSCs, which can be prevented by stabilizing extracellular matrix-deposited TGF-β in its inactive form by interacting with αv integrins (129, 130). In hepatocellular carcinoma (HCC), TGF-β plays a dual role, acting as a tumor-suppressor at early stages but contributing to tumor progression at late stages. Inhibition of TGF-β pathway may constitute an effective option for HCC treatment. However, the one-sided inhibition of TGF-β could have negative effects. Thus, it is mandatory to identify relevant biomarkers in TGF-β signaling with HCC (131). Furthermore, the increased bone resorption in liver disease may promote the release of TGF-β, and whether it may further aggravate the progression of liver disease needs further study. The findings of osteokines are summarised in **Table 2**.

**Table 2:** Osteokines involved in liver regulation.

Osteokines	The effect on bone	The effect on liver	Reference
FGF23	Disturb osteoblast differentiation and matrix mineralization	Elevate inflammatory cytokine expression in the liver	(32–34)
Osteocalcin	The abundant osteoblast-specific non-collagenous protein and a determinant of bone formation	Reduce the pathology of NAFLD	(35–38)
Calcitonin	Suppress bone resorption	Stimulate biliary proliferation/senescence and liver fibrosis	(39, 40)
Sclerostin	Suppress bone formation	Increase insulin resistance	(8, 41)
TGF-β	Being essential to osteogenesis	Accelerate liver fibrosis	(42, 43)
LCN 2	Promote osteogenic differentiation and osteogenesis	Accelerate NASH	(44, 45)

## 4 Discussion

**Figure 1:**
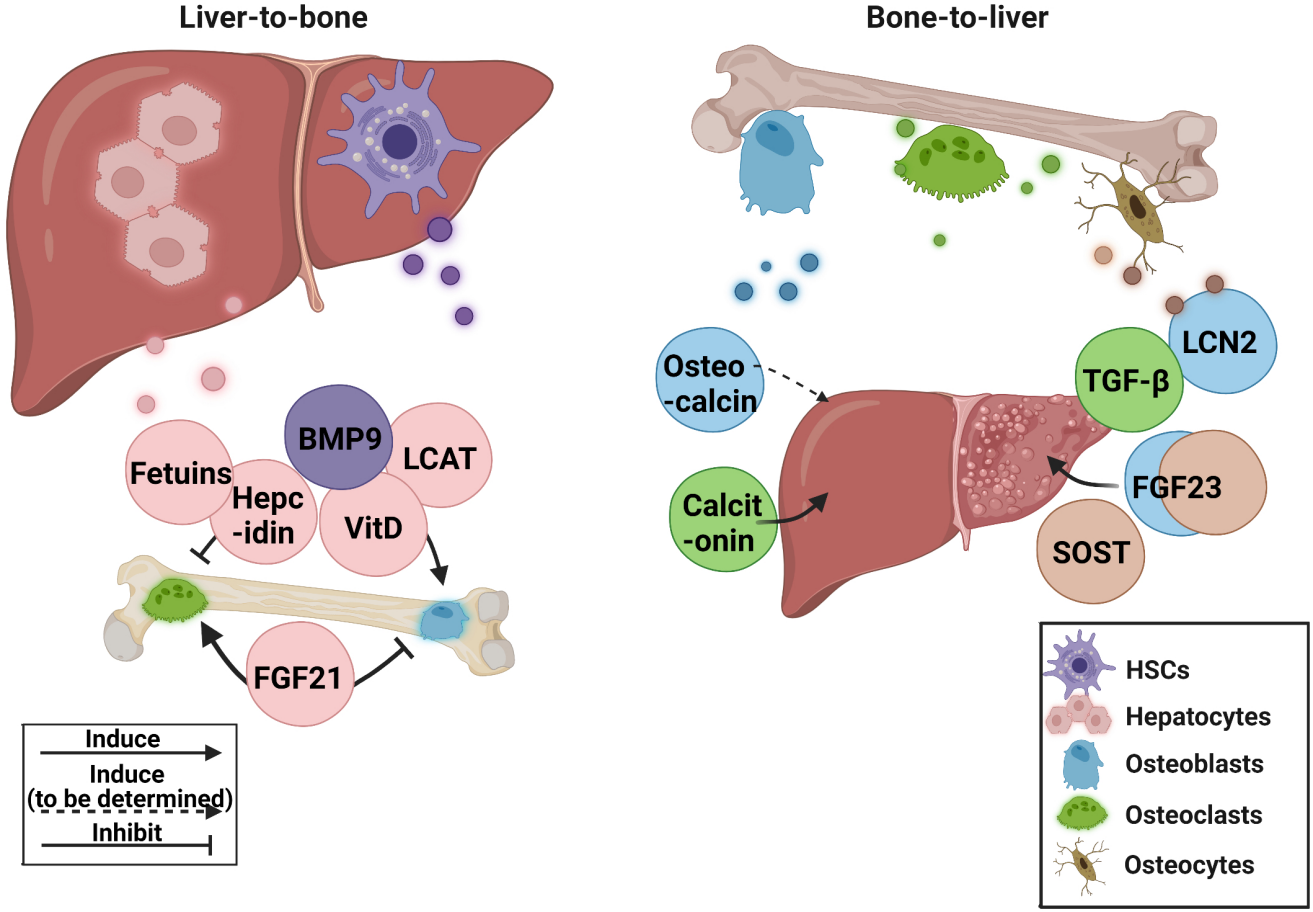
The current research status of related hepatokines and osteokines. BMP9, bone morphogenetic protein 9; FGF21, fibroblast growth factor 21; FGF23, fibroblast growth factor 23; HSCs, hepatic stellate cells; LCN2, lipocalin 2; LCAT, lecithin cholesterol acyltransferase; SOST, sclerostin; TGF-β, transforming growth factor β; VitD, vitamin D.

Increased bone fragility and decreased bone mass are common in patients with chronic liver disease. Abnormal liver metabolism is also related to the disruption of bone homeostasis. In recent years, some advancements have been made in liver-bone crosstalk. However, most of the mechanisms and clinical studies remain insufficient, and existing research has some contradictions. In this review, the role of hepatokines in bone homeostasis and osteokines in bone-liver crosstalk in preclinical and clinical research was discussed **Figure 1**.

The crucial roles of hepatokines FGF21 and BMP9 in controlling bone homeostasis, and osteokines calcitonin and SOST in regulating liver metabolism, are all elegantly demonstrated by transgenic animal studies and direct clinical investigations. Thus, these hepatokines and osteokines warrant further clinical translation as therapeutic targets or diagnostic markers for HOD or liver abnormalities in bone diseases. Although basic studies have revealed the potential contributions of hepatokines fetuins, hepcidin, and LCAT, and osteokine FGF23 to liver-bone crosstalk, the clinical significance still need to be verified in further studies. As for TGF-β, a widely distributed factor with dual regulatory effect on liver and bone, it is still worth exploring whether the massive release of bone-derived TGF-β causes additional burden on the liver directly or indirectly.

Other secretory components may be involved in the dialogue between the liver and bone in addition to hepatokines and osteokines. Currently, small extracellular vesicles derived from bone marrow mesenchymal stem cells exhibited the ability to promote bone and liver regeneration, and control immune responses (132). Exosome studies in liver-bone crosstalk are still lacking. Moreover, the release of inflammatory factors linked to bone disease can also cause damage to the liver microenvironment. These are worth exploring in future studies. Furthermore, multiple organ interactions, such as the liver-gut axis to the bone, and the bone-brain axis to the liver, may play an important role that cannot be overlooked. Some scattered clues are already observed. The Trimethylamine-N-Oxide, which is related to liver-associated bile acid metabolism and gut microbiota (133), could protect against BMD reduction (134). Besides, the gut-derived hormone FGF19 that belongs to the subfamily of FGF21 (135), could inhibit hepatic bile acid synthesis (136) and protect against obesity-induced bone loss (137). It might be a potential therapeutic target for the effects of obesity on skeletal muscle (138). The complicated relationship among the liver, gut, and bone may be better understood through research on those factors.

In addition, when it comes to clinical applications, several issues remain to be addressed. It is still difficult to differentiate the hepatic abnormalities between drug-related side effect and comorbid condition with aberrant bone metabolism. On the other hand, some biomarkers of bone metabolism, such as ALP, could be upregulated during liver injury, therefore, are not reliable in patients with hepatobiliary illnesses. Thus, it is desirable to develop reliable biomarkers for reflecting the pathological liver-bone crosstalk in the future.

With the growing recognition of the dynamic crosstalk between liver and bone and its contribution to pathological bone loss and liver abnormalities, we should not neglect the harmful signals from liver to bone when treating bone diseases or bone-related comorbidities in liver diseases, *i.e.*, HOD, and vice versa. Thus, preserving the physiological liver-bone crosstalk could be a promising strategy for improving liver and bone health, while interfering the pathological liver-bone crosstalk could be an alternative strategy for combating HODs. Importantly, the benefits and risks of targeting those hepatic or osteal factors involved in liver-bone crosstalk are currently ambiguous, which required to be evaluated by in-depth mechanistic studies and large-scale clinical trials in future.

## Author contributions

The construction of the main framework: ZL, JL. Collection of references: ZL, XW, NL, CZ, LC, FZ. References organization: ZL, XW. Literature analysis: ZL, XW, NL, JL. Manuscripts preparation: ZL, XW, JL. Manuscript revision: JL, GZ, AL. Funding: JL, AL. All authors approved the final version of the manuscript.

## References

[1] Maccabruni A Zaramella M Pedrotti L Lucanto S Quaglini S Mora R. Bone disorders in children and adolescents with chronic hcv infection. Clin cases Miner Bone Metab (2014) 11(2):99–104. doi: 10.11138/ccmbm/2014.11.2.099 25285135 PMC4172190

[2] Uchida S Miyaaki H Ichikawa T Taura N Miuma S Honda T. Risk factors for osteoporosis in patients with end-stage liver disease. BioMed Rep (2016) 5(5):629–33. doi: 10.3892/br.2016.764 PMC5103687 27882229

[3] Liu Z Han T Werner H Rosen CJ Schaffler MB Yakar S. Reduced serum igf-1 associated with hepatic osteodystrophy is a main determinant of low cortical but not trabecular bone mass. J Bone Miner Res (2018) 33(1):123–36. doi: 10.1002/jbmr.3290 PMC5771972 28902430

[4] Crosbie OM Freaney R McKenna MJ Hegarty JE. Bone density, vitamin d status, and disordered bone remodeling in end-stage chronic liver disease. Calcif Tissue Int (1999) 64(4):295–300. doi: 10.1007/s002239900622 10089221

[5] Tang Y Zhang M. Fibroblast growth factor 21 and bone homeostasis. BioMed J (2022). doi: 10.1016/j.bj.2022.07.002 PMC10345222 35850479

[6] Kurgan N McKee K Calleja M Josse AR Klentrou P. Cytokines, adipokines, and bone markers at rest and in response to plyometric exercise in obese vs normal weight adolescent females. Front Endocrinol (Lausanne) (2020) 11:531926. doi: 10.3389/fendo.2020.531926 33362710 PMC7759614

[7] Cao W Xu Y Shen Y Wang Y Ma X Bao Y. Serum fibroblast growth factor 23 level and liver fat content in mafld: A community-based cohort. Diabetes Metab Syndr Obes (2021) 14:4135–43. doi: 10.2147/DMSO.S328206 PMC8487847 34616166

[8] Takashi Y Kawanami D. The role of bone-derived hormones in glucose metabolism, diabetic kidney disease, and cardiovascular disorders. Int J Mol Sci (2022) 23(4):2376. doi: 10.3390/ijms23042376 PMC8879859 35216490

[9] Mosialou I Shikhel S Liu JM Maurizi A Luo N He Z. Mc4r-dependent suppression of appetite by bone-derived lipocalin 2. Nature (2017) 543(7645):385–90. doi: 10.1038/nature21697 PMC5975642 28273060

[10] Musso G Paschetta E Gambino R Cassader M Molinaro F. Interactions among bone, liver, and adipose tissue predisposing to diabesity and fatty liver. Trends Mol Med (2013) 19(9):522–35. doi: 10.1016/j.molmed.2013.05.006 23816817

[11] Giannini C Feldstein AE Santoro N Kim G Kursawe R Pierpont B. Circulating levels of fgf-21 in obese youth: Associations with liver fat content and markers of liver damage. J Clin Endocrinol Metab (2013) 98(7):2993–3000. doi: 10.1210/jc.2013-1250 23626003 PMC3701279

[12] Lin Z Gong Q Wu C Yu J Lu T Pan X. Dynamic change of serum Fgf21 levels in response to glucose challenge in human. J Clin Endocrinol Metab (2012) 97(7):E1224–8. doi: 10.1210/jc.2012-1132 22539584

[13] Yilmaz Y Eren F Yonal O Kurt R Aktas B Celikel CA. Increased serum Fgf21 levels in patients with nonalcoholic fatty liver disease. Eur J Clin Invest (2010) 40(10):887–92. doi: 10.1111/j.1365-2362.2010.02338.x 20624171

[14] Zarei M Pizarro-Delgado J Barroso E Palomer X Vazquez-Carrera M. Targeting Fgf21 for the treatment of nonalcoholic steatohepatitis. Trends Pharmacol Sci (2020) 41(3):199–208. doi: 10.1016/j.tips.2019.12.005 31980251

[15] Tillman EJ Rolph T. Fgf21: An emerging therapeutic target for non-alcoholic steatohepatitis and related metabolic diseases. Front Endocrinol (Lausanne) (2020) 11:601290. doi: 10.3389/fendo.2020.601290 33381084 PMC7767990

[16] Zheng Q Martin RC Shi X Pandit H Yu Y Liu X. Lack of Fgf21 promotes Nash-hcc transition *Via* hepatocyte-Tlr4-Il-17a signaling. Theranostics (2020) 10(22):9923–36. doi: 10.7150/thno.45988 PMC7481424 32929325

[17] Zhou L Song H-Y Gao L-L Yang L-Y Mu S Fu Q. Microrna−100−5p inhibits osteoclastogenesis and bone resorption by regulating fibroblast growth factor 21. Int J Mol Med (2019) 43(2):727–38. doi: 10.3892/ijmm.2018.4017 PMC6317653 30535435

[18] Wang X Wei W Krzeszinski JY Wang Y Wan Y. A liver-bone endocrine relay by Igfbp1 promotes osteoclastogenesis and mediates Fgf21-induced bone resorption. Cell Metab (2015) 22(5):811–24. doi: 10.1016/j.cmet.2015.09.010 PMC4635071 26456333

[19] Wei W Dutchak PA Wang X Ding X Wang X Bookout AL. Fibroblast growth factor 21 promotes bone loss by potentiating the effects of peroxisome proliferator-activated receptor gamma. Proc Natl Acad Sci U.S.A. (2012) 109(8):3143–8. doi: 10.1073/pnas.1200797109 PMC3286969 22315431

[20] Yang Z Li P Shang Q Wang Y He J Ge S. Crispr-mediated Bmp9 ablation promotes liver steatosis via the down-regulation of pparalpha expression. Sci Adv (2020) 6(48):eabc5022. doi: 10.1126/sciadv.abc5022 PMC7695473 33246954

[21] Zhou YM Yang YY Jing YX Yuan TJ Sun LH Tao B. Bmp9 reduces bone loss in ovariectomized mice by dual regulation of bone remodeling. J Bone Miner Res (2020) 35(5):978–93. doi: 10.1002/jbmr.3957 31914211

[22] de la Puente Yague M Collado Yurrita L Ciudad Cabanas MJ Cuadrado Cenzual MA. Role of vitamin d in athletes and their performance: Current concepts and new trends. Nutrients (2020) 12(2):579. doi: 10.3390/nu12020579 PMC7071499 32102188

[23] Zuniga S Firrincieli D Housset C Chignard N. Vitamin d and the vitamin d receptor in liver pathophysiology. Clin Res Hepatol Gastroenterol (2011) 35(4):295–302. doi: 10.1016/j.clinre.2011.02.003 21440524

[24] Innao V Allegra A Ginaldi L Pioggia G De Martinis M Musolino C. Reviewing the significance of vitamin d substitution in monoclonal gammopathies. Int J Mol Sci (2021) 22(9):4922. doi: 10.3390/ijms22094922 PMC8124934 34066482

[25] Rittig K Thamer C Haupt A Machann J Peter A Balletshofer B. High plasma fetuin-a is associated with increased carotid intima-media thickness in a middle-aged population. Atherosclerosis (2009) 207(2):341–2. doi: 10.1016/j.atherosclerosis.2009.05.018 19615685

[26] Liu S Xiao J Zhao Z Wang M Wang Y Xin Y. Systematic review and meta-analysis of circulating fetuin-a levels in nonalcoholic fatty liver disease. J Clin Transl Hepatol (2021) 9(1):3–14. doi: 10.14218/JCTH.2020.00081 33604250 PMC7868693

[27] Jablonski H Polan C Wedemeyer C Hilken G Schlepper R Bachmann HS. A single intraperitoneal injection of bovine fetuin-a attenuates bone resorption in a murine calvarial model of particle-induced osteolysis. Bone (2017) 105:262–8. doi: 10.1016/j.bone.2017.09.006 28942123

[28] Chen H Zhao W Yan X Huang T Yang A. Overexpression of hepcidin alleviates steatohepatitis and fibrosis in a diet-induced nonalcoholic steatohepatitis. J Clin Transl Hepatol (2022) 10(4):577–88. doi: 10.14218/JCTH.2021.00289 PMC9396326 36062292

[29] Guo HH Xiong L Pan JX Lee D Liu K Ren X. Hepcidin contributes to Swedish mutant app-induced osteoclastogenesis and trabecular bone loss. Bone Res (2021) 9(1):31. doi: 10.1038/s41413-021-00146-0 34108442 PMC8190093

[30] Qiao M Wu HY Li FE Jiang SW Xiong YZ Deng CY. Molecular characterization, expression profile and association analysis with carcass traits of porcine lcat gene. Mol Biol Rep (2010) 37(5):2227–34. doi: 10.1007/s11033-009-9709-x 19672691

[31] Triantaphyllidou IE Kalyvioti E Karavia E Lilis I Kypreos KE Papachristou DJ. Perturbations in the hdl metabolic pathway predispose to the development of osteoarthritis in mice following long-term exposure to Western-type diet. Osteoarthritis Cartilage (2013) 21(2):322–30. doi: 10.1016/j.joca.2012.11.003 23151457

[32] Zhang DD Wu YF Chen WX Xu Y Liu SY Luo HH. C-type natriuretic peptide attenuates renal osteodystrophy through inhibition of fgf-23/Mapk signaling. Exp Mol Med (2019) 51(7):1–18. doi: 10.1038/s12276-019-0265-8 PMC6802631 31263178

[33] Kumar P Liu Y Shen Y Maher JJ Cingolani F Czaja MJ. Mouse liver injury induces hepatic macrophage Fgf23 production. PloS One (2022) 17(3):e0264743. doi: 10.1371/journal.pone.0264743 35231062 PMC8887750

[34] Richter B Faul C. Fgf23 actions on target tissues-with and without klotho. Front Endocrinol (Lausanne) (2018) 9:189. doi: 10.3389/fendo.2018.00189 29770125 PMC5940753

[35] Zhang M Nie X Yuan Y Wang Y Ma X Yin J. Osteocalcin alleviates nonalcoholic fatty liver disease in mice through Gprc6a. Int J Endocrinol (2021) 2021:9178616. doi: 10.1155/2021/9178616 33531899 PMC7834799

[36] Teng B Huang C Cheng C-L Udduttula A Yu X-F Liu C. Newly identified peptide hormone inhibits intestinal fat absorption and improves nafld through its receptor Gprc6a. J Hepatol (2020) 73(2):383–93. doi: 10.1016/j.jhep.2020.02.026 32147363

[37] DeMambro VE Clemmons DR Horton LG Bouxsein ML Wood TL Beamer WG. Gender-specific changes in bone turnover and skeletal architecture in igfbp-2-Null mice. Endocrinology (2008) 149(5):2051–61. doi: 10.1210/en.2007-1068 PMC2329262 18276763

[38] Ducy P Desbois C Boyce B Pinero G Story B Dunstan C. Increased bone formation in osteocalcin-deficient mice. Nature (1996) 382(6590):448–52. doi: 10.1038/382448a0 8684484

[39] Davey RA Turner AG McManus JF Chiu WS Tjahyono F Moore AJ. Calcitonin receptor plays a physiological role to protect against hypercalcemia in mice. J Bone Miner Res (2008) 23(8):1182–93. doi: 10.1359/jbmr.080310 PMC2680171 18627265

[40] Mancinelli R Ceci L Kennedy L Francis H Meadows V Chen L. The effects of taurocholic acid on biliary damage and liver fibrosis are mediated by calcitonin-Gene-Related peptide signaling. Cells (2022) 11(9):1591. doi: 10.3390/cells11091591 PMC9104610 35563897

[41] Liu S Zhou J Tang W Jiang X Rowe DW Quarles LD. Pathogenic role of Fgf23 in hyp mice. Am J Physiol Endocrinol Metab (2006) 291(1):E38–49. doi: 10.1152/ajpendo.00008.2006 16449303

[42] Shen GY Ren H Shang Q Zhao WH Zhang ZD Yu X. Let-7f-5p regulates Tgfbr1 in glucocorticoid-inhibited osteoblast differentiation and ameliorates glucocorticoid-induced bone loss. Int J Biol Sci (2019) 15(10):2182–97. doi: 10.7150/ijbs.33490 PMC6775285 31592234

[43] Yoshida K Murata M Yamaguchi T Matsuzaki K. Tgf-Beta/Smad signaling during hepatic fibro-carcinogenesis (Review). Int J Oncol (2014) 45(4):1363–71. doi: 10.3892/ijo.2014.2552 PMC4151811 25050845

[44] Yin C Jia X Zhao Q Zhao Z Wang J Zhang Y. Transcription factor 7-like 2 promotes osteogenic differentiation and boron-induced bone repair *Via* lipocalin 2. Mater Sci Eng C Mater Biol Appl (2020) 110:110671. doi: 10.1016/j.msec.2020.110671 32204099

[45] Ye D Yang K Zang S Lin Z Chau HT Wang Y. Lipocalin-2 mediates non-alcoholic steatohepatitis by promoting neutrophil-macrophage crosstalk *Via* the induction of Cxcr2. J Hepatol (2016) 65(5):988–97. doi: 10.1016/j.jhep.2016.05.041 27266617

[46] Talukdar S Zhou Y Li D Rossulek M Dong J Somayaji V. A long-acting Fgf21 molecule, pf-05231023, decreases body weight and improves lipid profile in non-human primates and type 2 diabetic subjects. Cell Metab (2016) 23(3):427–40. doi: 10.1016/j.cmet.2016.02.001 26959184

[47] Gallego-Escuredo JM Lamarca MK Villarroya J Domingo JC Mateo MG Gutierrez MDM. High Fgf21 levels are associated with altered bone homeostasis in hiv-1-Infected patients. Metabolism (2017) 71:163–70. doi: 10.1016/j.metabol.2017.03.014 28521869

[48] Lee SY Fam KD Chia KL Yap MMC Goh J Yeo KP. Age-related bone loss is associated with Fgf21 but not Igfbp1 in healthy adults. Exp Physiol (2020) 105(4):622–31. doi: 10.1113/EP088351 31977105

[49] Pye SR Almusalam B Boonen S Vanderschueren D Borghs H Gielen E. Influence of insulin-like growth factor binding protein (Igfbp)-1 and igfbp-3 on bone health: Results from the European Male ageing study. Calcif Tissue Int (2011) 88(6):503–10. doi: 10.1007/s00223-011-9484-2 PMC3920365 21503646

[50] Lee P Linderman J Smith S Brychta RJ Perron R Idelson C. Fibroblast growth factor 21 (Fgf21) and bone: Is there a relationship in humans? Osteoporos Int (2013) 24(12):3053–7. doi: 10.1007/s00198-013-2464-9 PMC6314482 23912560

[51] Hu W He J Fu W Wang C Yue H Gu J. Fibroblast growth factor 21 is associated with bone mineral density, but not with bone turnover markers and fractures in Chinese postmenopausal women. J Clin Densitom (2019) 22(2):179–84. doi: 10.1016/j.jocd.2018.08.005 30228048

[52] Akduman F Siklar Z Ozsu E Dogan O Kir MK Berberoglu M. Fgf21 levels and bone mineral density in metabolically healthy and metabolically unhealthy obese children. J Clin Res Pediatr Endocrinol (2022). 14 (4) 433–443 doi: 10.4274/jcrpe.galenos.2022.2022-1-15 PMC9724058 35859690

[53] Song T Huang D Song D. The potential regulatory role of Bmp9 in inflammatory responses. Genes Dis (2022) 9(6):1566–78. doi: 10.1016/j.gendis.2021.08.010 PMC9485205 36157503

[54] Um J-H Park S-Y Hur JH Lee H-Y Jeong K-H Cho Y. Bone morphogenic protein 9 is a novel thermogenic hepatokine secreted in response to cold exposure. Metabolism: Clin Exp (2022) 129:155139. doi: 10.1016/j.metabol.2022.155139 35063533

[55] Desroches-Castan A Tillet E Ricard N Ouarne M Mallet C Belmudes L. Bone morphogenetic protein 9 is a paracrine factor controlling liver sinusoidal endothelial cell fenestration and protecting against hepatic fibrosis. Hepatology (2019) 70(4):1392–408. doi: 10.1002/hep.30655 30964206

[56] Tang N Rao S Ying Y Huang Y. New insights into Bmp9 signaling in organ fibrosis. Eur J Pharmacol (2020) 882:173291. doi: 10.1016/j.ejphar.2020.173291 32574673

[57] Chen H Brady Ridgway J Sai T Lai J Warming S Chen H. Context-dependent signaling defines roles of Bmp9 and Bmp10 in embryonic and postnatal development. Proc Natl Acad Sci U.S.A. (2013) 110(29):11887–92. doi: 10.1073/pnas.1306074110 PMC3718114 23812757

[58] Chiba N Noguchi Y Seong CH Ohnishi T Matsuguchi T. Egr1 plays an important role in Bmp9-mediated osteoblast differentiation by promoting Smad1/5 phosphorylation. FEBS Lett (2022) 596(13):1720–32. doi: 10.1002/1873-3468.14407 35594155

[59] Wang X Huang J Huang F Zong JC Tang X Liu Y. Bone morphogenetic protein 9 stimulates callus formation in osteoporotic rats during fracture healing. Mol Med Rep (2017) 15(5):2537–45. doi: 10.3892/mmr.2017.6302 PMC5428899 28447742

[60] Liu Y Liu Y Zhang R Wang X Huang F Yan Z. All-trans retinoic acid modulates bone morphogenic protein 9-induced osteogenesis and adipogenesis of preadipocytes through Bmp/Smad and Wnt/Beta-catenin signaling pathways. Int J Biochem Cell Biol (2014) 47:47–56. doi: 10.1016/j.biocel.2013.11.018 24300824

[61] Xu JZ Zhou YM Zhang LL Chen XJ Yang YY Zhang D. Bmp9 reduces age-related bone loss in mice by inhibiting osteoblast senescence through Smad1-Stat1-P21 axis. Cell Death Discovery (2022) 8(1):254. doi: 10.1038/s41420-022-01048-8 35523787 PMC9076651

[62] Wang Y Feng Q Ji C Liu X Li L Luo J. Runx3 plays an important role in mediating the Bmp9-induced osteogenic differentiation of mesenchymal stem cells. Int J Mol Med (2017) 40(6):1991–9. doi: 10.3892/ijmm.2017.3155 29039519

[63] Zhou Y Liu C He J Dong L Zhu H Zhang B. Klf2(+) stemness maintains human mesenchymal stem cells in bone regeneration. Stem Cells (2020) 38(3):395–409. doi: 10.1002/stem.3120 31721356

[64] Stallhofer J Veith L Diegelmann J Probst P Brand S Schnitzler F. Iron deficiency in inflammatory bowel disease is associated with low levels of vitamin d modulating serum hepcidin and intestinal ceruloplasmin expression. Clin Transl Gastroenterol (2022) 13(1):e00450. doi: 10.14309/ctg.0000000000000450 35029158 PMC8806373

[65] Ehnert S Aspera-Werz RH Ruoss M Dooley S Hengstler JG Nadalin S. Hepatic osteodystrophy-molecular mechanisms proposed to favor its development. Int J Mol Sci (2019) 20(10):2555. doi: 10.3390/ijms20102555 PMC6566554 31137669

[66] Xiao K Zhang DC Hu Y Song LC Xu JQ He WX. Potential roles of vitamin d binding protein in attenuating liver injury in sepsis. Mil Med Res (2022) 9(1):4. doi: 10.1186/s40779-022-00365-4 35057868 PMC8772176

[67] Shirvani A Kalajian TA Song A Holick MF. Disassociation of vitamin d's calcemic activity and non-calcemic genomic activity and individual responsiveness: A randomized controlled double-blind clinical trial. Sci Rep (2019) 9(1):17685. doi: 10.1038/s41598-019-53864-1 31776371 PMC6881448

[68] Cen C Wang W Yu S Tang X Liu J Liu Y. Development and validation of a clinical and laboratory-based nomogram to predict nonalcoholic fatty liver disease. Hepatol Int (2020) 14(5):808–16. doi: 10.1007/s12072-020-10065-7 32572817

[69] Pereira F Azevedo R Linhares M Pinto J Leitao C Caldeira A. Hepatic osteodystrophy in cirrhosis due to alcohol-related liver disease. Rev Esp Enferm Dig (2021) 113(8):563–9. doi: 10.17235/reed.2020.7301/2020 33267594

[70] Zhang Y Gao X Liu T Gao P Li H Liu N. Association between osteoporosis and hepatitis b cirrhosis: A case-control study. Afr Health Sci (2020) 20(4):1610–6. doi: 10.4314/ahs.v20i4.13 PMC8351827 34394221

[71] Wang F So KF Xiao J Wang H. Organ-organ communication: The liver's perspective. Theranostics (2021) 11(7):3317–30. doi: 10.7150/thno.55795 PMC7847667 33537089

[72] Kan C Yang J Fan H Dai Y Wang X Chen R. Fetuin-a is an immunomodulator and a potential therapeutic option in Bmp4-dependent heterotopic ossification and associated bone mass loss. Bone Res (2022) 10(1):62. doi: 10.1038/s41413-022-00232-x 36289197 PMC9605967

[73] Sritara C Thakkinstian A Ongphiphadhanakul B Chailurkit L Chanprasertyothin S Ratanachaiwong W. Causal relationship between the ahsg gene and bmd through fetuin-a and bmi: Multiple mediation analysis. Osteoporos Int (2014) 25(5):1555–62. doi: 10.1007/s00198-014-2634-4 24570294

[74] Chailurkit L Kruavit A Rajatanavin R Ongphiphadhanakul B. The relationship of fetuin-a and lactoferrin with bone mass in elderly women. Osteoporos Int (2011) 22(7):2159–64. doi: 10.1007/s00198-010-1439-3 20963400

[75] FinkHABuzkovaPGarimellaPSMukamalKJCauleyJAKizerJR. Association of fetuin-a with incident fractures in community-dwelling older adults: The cardiovascular health study. J Bone Miner Res (2015) 30(8):1394–402. doi: 10.1002/jbmr.2475 25656814

[76] Meex RC Hoy AJ Morris A Brown RD Lo JCY Burke M. Fetuin b is a secreted hepatocyte factor linking steatosis to impaired glucose metabolism. Cell Metab (2015) 22(6):1078–89. doi: 10.1016/j.cmet.2015.09.023 26603189

[77] Wang D Liu Y Liu S Lin L Liu C Shi X. Serum fetuin-b is positively associated with intrahepatic triglyceride content and increases the risk of insulin resistance in obese Chinese adults: A cross-sectional study. J Diabetes (2018) 10(7):581–8. doi: 10.1111/1753-0407.12632 29194974

[78] Xu ZH He J Zhang X Xie H Zhou ZH Sun YW. Serum level of fetuin b is associated with osteoporosis: A 4-year prospective study in China. Clin Invest Med (2018) 41(1):E25–30. doi: 10.25011/cim.v41i1.29460 29603688

[79] Enns CA Jue S Zhang AS. Hepatocyte neogenin is required for hemojuvelin-mediated hepcidin expression and iron homeostasis in mice. Blood (2021) 138(6):486–99. doi: 10.1182/blood.2020009485 PMC8370464 33824974

[80] Varghese J Varghese James J Karthikeyan M Rasalkar K Raghavan R Sukumaran A. Iron homeostasis is dysregulated, but the iron-hepcidin axis is functional, in chronic liver disease. J Trace Elem Med Biol (2020) 58:126442. doi: 10.1016/j.jtemb.2019.126442 31835128

[81] Ma J Wang A Zhang H Liu B Geng Y Xu Y. Iron overload induced osteocytes apoptosis and led to bone loss in hepcidin(-/-) mice through increasing sclerostin and Rankl/Opg. Bone (2022) 164:116511. doi: 10.1016/j.bone.2022.116511 35933095

[82] Min HK Sung SA Oh YK Kim YH Chung W Park SK. Hepcidin, iron indices and bone mineral metabolism in non-dialysis chronic kidney disease. Nephrol Dial Transplant (2020) 35(1):147–54. doi: 10.1093/ndt/gfy235 30053139

[83] Zaidi M Yuen T Iqbal J. Reverse cholesterol transport and hepatic osteodystrophy. Cell Metab (2022) 34(3):347–9. doi: 10.1016/j.cmet.2022.02.007 PMC11658025 35235770

[84] Laurenzi T Parravicini C Palazzolo L Guerrini U Gianazza E Calabresi L. Rhdl modeling and the anchoring mechanism of lcat activation. J Lipid Res (2021) 62:100006. doi: 10.1194/jlr.RA120000843 33518511 PMC7859856

[85] Privitera G Spadaro L Marchisello S Fede G Purrello F. Abnormalities of lipoprotein levels in liver cirrhosis: Clinical relevance. Dig Dis Sci (2018) 63(1):16–26. doi: 10.1007/s10620-017-4862-x 29177578

[86] Nass KJ van den Berg EH Gruppen EG Dullaart RPF. Plasma Lecithin:Cholesterol acyltransferase and phospholipid transfer protein activity independently associate with nonalcoholic fatty liver disease. Eur J Clin Invest (2018) 48(9):e12988. doi: 10.1111/eci.12988 29947103

[87] Lu K Shi TS Shen SY Shi Y Gao HL Wu J. Defects in a liver-bone axis contribute to hepatic osteodystrophy disease progression. Cell Metab (2022) 34(3):441–57 e7. doi: 10.1016/j.cmet.2022.02.006 35235775

[88] Urakawa I Yamazaki Y Shimada T Iijima K Hasegawa H Okawa K. Klotho converts canonical fgf receptor into a specific receptor for Fgf23. Nature (2006) 444(7120):770–4. doi: 10.1038/nature05315 17086194

[89] Kinoshita Y Takashi Y Ito N Ikegawa S Mano H Ushiku T. Ectopic expression of klotho in fibroblast growth factor 23 (Fgf23)-producing tumors that cause tumor-induced Rickets/Osteomalacia (Tio). Bone Rep (2019) 10:100192. doi: 10.1016/j.bonr.2018.100192 30627598 PMC6321977

[90] Zisman AL Wolf M. Recent advances in the rapidly evolving field of fibroblast growth factor 23 in chronic kidney disease. Curr Opin Nephrol Hypertens (2010) 19(4):335–42. doi: 10.1097/mnh.0b013e328338f536 20583336

[91] Trombetti A Al-Daghri N Brandi ML Cannata-Andia JB Cavalier E Chandran M. Interdisciplinary management of Fgf23-related phosphate wasting syndromes: A consensus statement on the evaluation, diagnosis and care of patients with X-linked hypophosphataemia. Nat Rev Endocrinol (2022) 18(6):366–84. doi: 10.1038/s41574-022-00662-x 35484227

[92] Mameli C Sangiorgio A Colombo V Gambino M Spaccini L Cattaneo E. Autosomal dominant hypophosphatemic rickets: A case report and review of the literature. Int J Environ Res Public Health (2021) 18(16):8771. doi: 10.3390/ijerph18168771 PMC8392413 34444516

[93] Edmonston D Wolf M. Fgf23 at the crossroads of phosphate, iron economy and erythropoiesis. Nat Rev Nephrol (2020) 16(1):7–19. doi: 10.1038/s41581-019-0189-5 31519999

[94] Singh S Grabner A Yanucil C Schramm K Czaya B Krick S. Fibroblast growth factor 23 directly targets hepatocytes to promote inflammation in chronic kidney disease. Kidney Int (2016) 90(5):985–96. doi: 10.1016/j.kint.2016.05.019 PMC5065745 27457912

[95] Mattinzoli D Ikehata M Tsugawa K Alfieri CM Dongiovanni P Trombetta E. Fgf23 and fetuin-a interaction in the liver and in the circulation. Int J Biol Sci (2018) 14(6):586–98. doi: 10.7150/ijbs.23256 PMC6001652 29904273

[96] He X Shen Y Ma X Ying L Peng J Pan X. The association of serum Fgf23 and non-alcoholic fatty liver disease is independent of vitamin d in type 2 diabetes patients. Clin Exp Pharmacol Physiol (2018) 45(7):668–74. doi: 10.1111/1440-1681.12933 29574933

[97] Mao H Li L Fan Q Angelini A Saha PK Coarfa C. Endothelium-specific depletion of Lrp1 improves glucose homeostasis through inducing osteocalcin. Nat Commun (2021) 12(1):5296. doi: 10.1038/s41467-021-25673-6 34489478 PMC8421392

[98] Lambert LJ Challa AK Niu A Zhou L Tucholski J Johnson MS. Increased trabecular bone and improved biomechanics in an osteocalcin-null rat model created by Crispr/Cas9 technology. Dis Model Mech (2016) 9(10):1169–79. doi: 10.1242/dmm.025247 PMC5087831 27483347

[99] Caluwe R Pyfferoen L De Boeck K De Vriese AS. The effects of vitamin K supplementation and vitamin K antagonists on progression of vascular calcification: Ongoing randomized controlled trials. Clin Kidney J (2016) 9(2):273–9. doi: 10.1093/ckj/sfv146 PMC4792621 26985380

[100] Cipriani C Colangelo L Santori R Renella M Mastrantonio M Minisola S. The interplay between bone and glucose metabolism. Front Endocrinol (Lausanne) (2020) 11:122. doi: 10.3389/fendo.2020.00122 32265831 PMC7105593

[101] Xia M Rong S Zhu X Yan H Chang X Sun X. Osteocalcin and non-alcoholic fatty liver disease: Lessons from two population-based cohorts and animal models. J Bone Miner Res (2021) 36(4):712–28. doi: 10.1002/jbmr.4227 33270924

[102] Du J Zhang M Lu J Zhang X Xiong Q Xu Y. Osteocalcin improves nonalcoholic fatty liver disease in mice through activation of Nrf2 and inhibition of jnk. Endocrine (2016) 53(3):701–9. doi: 10.1007/s12020-016-0926-5 26994931

[103] Gupte AA Sabek OM Fraga D Minze LJ Nishimoto SK Liu JZ. Osteocalcin protects against nonalcoholic steatohepatitis in a mouse model of metabolic syndrome. Endocrinology (2014) 155(12):4697–705. doi: 10.1210/en.2014-1430 PMC5393336 25279794

[104] Wu XL Zou XY Zhang M Hu HQ Wei XL Jin ML. Osteocalcin prevents insulin resistance, hepatic inflammation, and activates autophagy associated with high-fat diet-induced fatty liver hemorrhagic syndrome in aged laying hens. Poult Sci (2021) 100(1):73–83. doi: 10.1016/j.psj.2020.10.022 33357709 PMC7772703

[105] Wang N Wang Y Chen X Zhang W Chen Y Xia F. Bone turnover markers and probable advanced nonalcoholic fatty liver disease in middle-aged and elderly men and postmenopausal women with type 2 diabetes. Front Endocrinol (Lausanne) (2019) 10:926. doi: 10.3389/fendo.2019.00926 32063885 PMC6999074

[106] Sinn DH Gwak GY Rhee SY Cho J Son HJ Paik YH. Association between serum osteocalcin levels and non-alcoholic fatty liver disease in women. Digestion (2015) 91(2):150–7. doi: 10.1159/000369789 25677815

[107] Naot D Musson DS Cornish J. The activity of peptides of the calcitonin family in bone. Physiol Rev (2019) 99(1):781–805. doi: 10.1152/physrev.00066.2017 30540227

[108] Garelja ML Bower RL Brimble MA Chand S Harris PWR Jamaluddin MA. Pharmacological characterisation of mouse calcitonin and calcitonin receptor-like receptors reveals differences compared with human receptors. Br J Pharmacol (2022) 179(3):416–34. doi: 10.1111/bph.15628 PMC8776895 34289083

[109] Karsdal MA Henriksen K Arnold M Christiansen C. Calcitonin: A drug of the past or for the future? physiologic inhibition of bone resorption while sustaining osteoclast numbers improves bone quality. BioDrugs (2008) 22(3):137–44. doi: 10.2165/00063030-200822030-00001 18481897

[110] Nakamura M Nomura S Yamakawa T Kono R Maeno A Ozaki T. Endogenous calcitonin regulates lipid and glucose metabolism in diet-induced obesity mice. Sci Rep (2018) 8(1):17001. doi: 10.1038/s41598-018-35369-5 30451912 PMC6242993

[111] Larsen AT Sonne N Andreassen KV Karsdal MA Henriksen K. The calcitonin receptor plays a major role in glucose regulation as a function of dual amylin and calcitonin receptor agonist therapy. J Pharmacol Exp Ther (2020) 374(1):74–83. doi: 10.1124/jpet.119.263392 32317372

[112] Larsen AT Gydesen S Sonne N Karsdal MA Henriksen K. The dual amylin and calcitonin receptor agonist kbp-089 and the glp-1 receptor agonist liraglutide act complimentarily on body weight reduction and metabolic profile. BMC Endocr Disord (2021) 21(1):10. doi: 10.1186/s12902-020-00678-2 33413317 PMC7791885

[113] Bracq S Machairas M Clement B Pidoux E Andreoletti M Moukhtar MS. Calcitonin gene expression in normal human liver. FEBS Lett (1993) 331(1-2):15–8. doi: 10.1016/0014-5793(93)80288-6 8405394

[114] McClung MR Grauer A Boonen S Bolognese MA Brown JP Diez-Perez A. Romosozumab in postmenopausal women with low bone mineral density. N Engl J Med (2014) 370(5):412–20. doi: 10.1056/NEJMoa1305224 24382002

[115] Langdahl BL Libanati C Crittenden DB Bolognese MA Brown JP Daizadeh NS. Romosozumab (Sclerostin monoclonal antibody) versus teriparatide in postmenopausal women with osteoporosis transitioning from oral bisphosphonate therapy: A randomised, open-label, phase 3 trial. Lancet (2017) 390(10102):1585–94. doi: 10.1016/S0140-6736(17)31613-6 28755782

[116] Saag KG Petersen J Brandi ML Karaplis AC Lorentzon M Thomas T. Romosozumab or alendronate for fracture prevention in women with osteoporosis. N Engl J Med (2017) 377(15):1417–27. doi: 10.1056/NEJMoa1708322 28892457

[117] Kim SP Frey JL Li Z Kushwaha P Zoch ML Tomlinson RE. Sclerostin influences body composition by regulating catabolic and anabolic metabolism in adipocytes. Proc Natl Acad Sci United States America (2017) 114(52):E11238–E47. doi: 10.1073/pnas.1707876115 PMC5748171 29229807

[118] Martin Gonzalez C Fernandez Rodriguez CM Abreu Gonzalez P Garcia Rodriguez A Alvisa Negrin JC Cabanas Perales E. Sclerostin in excessive drinkers: Relationships with liver function and body composition. Nutrients (2022) 14(13):2574. doi: 10.3390/nu14132574 PMC9268012 35807755

[119] Rhee Y Kim WJ Han KJ Lim SK Kim SH. Effect of liver dysfunction on circulating sclerostin. J Bone Miner Metab (2014) 32(5):545–9. doi: 10.1007/s00774-013-0524-z 24126695

[120] Wakolbinger R Muschitz C Wallwitz J Bodlaj G Feichtinger X Schanda JE. Serum levels of sclerostin reflect altered bone microarchitecture in patients with hepatic cirrhosis. Wien Klin Wochenschr (2020) 132(1-2):19–26. doi: 10.1007/s00508-019-01595-8 31912287 PMC6978289

[121] Zhou F Wang Y Li Y Tang M Wan S Tian H. Decreased sclerostin secretion in humans and mice with nonalcoholic fatty liver disease. Front Endocrinol (Lausanne) (2021) 12:707505. doi: 10.3389/fendo.2021.707505 34421825 PMC8374147

[122] Yan QW Yang Q Mody N Graham TE Hsu CH Xu Z. The adipokine lipocalin 2 is regulated by obesity and promotes insulin resistance. Diabetes (2007) 56(10):2533–40. doi: 10.2337/db07-0007 17639021

[123] Kim KE Lee J Shin HJ Jeong EA Jang HM Ahn YJ. Lipocalin-2 activates hepatic stellate cells and promotes nonalcoholic steatohepatitis in high-fat diet-fed Ob/Ob mice. Hepatology (2022). 77(3):889–901 doi: 10.1002/hep.32569 PMC9936980 35560370

[124] Wang Y Lam KS Kraegen EW Sweeney G Zhang J Tso AW. Lipocalin-2 is an inflammatory marker closely associated with obesity, insulin resistance, and hyperglycemia in humans. Clin Chem (2007) 53(1):34–41. doi: 10.1373/clinchem.2006.075614 17040956

[125] Abella V Scotece M Conde J Gomez R Lois A Pino J. The potential of lipocalin-2/Ngal as biomarker for inflammatory and metabolic diseases. Biomarkers (2015) 20(8):565–71. doi: 10.3109/1354750X.2015.1123354 PMC4819811 26671823

[126] Chen J Argemi J Odena G Xu MJ Cai Y Massey V. Hepatic lipocalin 2 promotes liver fibrosis and portal hypertension. Sci Rep (2020) 10(1):15558. doi: 10.1038/s41598-020-72172-7 32968110 PMC7512007

[127] Mishra J Mori K Ma Q Kelly C Yang J Mitsnefes M. Amelioration of ischemic acute renal injury by neutrophil gelatinase-associated lipocalin. J Am Soc Nephrol (2004) 15(12):3073–82. doi: 10.1097/01.ASN.0000145013.44578.45 15579510

[128] Lamora A Talbot J Mullard M Brounais-Le Royer B Redini F Verrecchia F. Tgf-beta signaling in bone remodeling and osteosarcoma progression. J Clin Med (2016) 5(11):96. doi: 10.3390/jcm5110096 PMC5126793 27827889

[129] Fan W Liu T Chen W Hammad S Longerich T Hausser I. Ecm1 prevents activation of transforming growth factor beta, hepatic stellate cells, and fibrogenesis in mice. Gastroenterology (2019) 157(5):1352–67 e13. doi: 10.1053/j.gastro.2019.07.036 31362006

[130] Chen M Liu J Yang W Ling W. Lipopolysaccharide mediates hepatic stellate cell activation by regulating autophagy and retinoic acid signaling. Autophagy (2017) 13(11):1813–27. doi: 10.1080/15548627.2017.1356550 PMC5788469 29160747

[131] Gonzalez-Sanchez E Vaquero J Fernandez-Barrena MG Lasarte JJ Avila MA Sarobe P. The tgf-beta pathway: A pharmacological target in hepatocellular carcinoma? Cancers (Basel) (2021) 13(13):3248. doi: 10.3390/cancers13133248 PMC8268320 34209646

[132] Liu L Guo S Shi W Liu Q Huo F Wu Y. Bone marrow mesenchymal stem cell-derived small extracellular vesicles promote periodontal regeneration. Tissue Eng Part A (2021) 27(13-14):962–76. doi: 10.1089/ten.TEA.2020.0141 32962564

[133] Chen ML Yi L Zhang Y Zhou X Ran L Yang J. Resveratrol attenuates trimethylamine-N-Oxide (Tmao)-induced atherosclerosis by regulating tmao synthesis and bile acid metabolism *Via* remodeling of the gut microbiota. mBio (2016) 7(2):e02210–15. doi: 10.1128/mBio.02210-15 PMC4817264 27048804

[134] Zhou T Heianza Y Chen Y Li X Sun D DiDonato JA. Circulating gut microbiota metabolite trimethylamine n-oxide (Tmao) and changes in bone density in response to weight loss diets: The pounds lost trial. Diabetes Care (2019) 42(8):1365–71. doi: 10.2337/dc19-0134 PMC6647048 31332027

[135] Chen H Li J Zhang D Zhou X Xie J. Role of the fibroblast growth factor 19 in the skeletal system. Life Sci (2021) 265:118804. doi: 10.1016/j.lfs.2020.118804 33245964

[136] Henriksson E Andersen B. Fgf19 and Fgf21 for the treatment of Nash-two sides of the same coin? differential and overlapping effects of Fgf19 and Fgf21 from mice to human. Front Endocrinol (Lausanne) (2020) 11:601349. doi: 10.3389/fendo.2020.601349 33414764 PMC7783467

[137] Guo A Li K Tian HC Tao BL Xiao Q Jiang DM. Fgf19 protects against obesity-induced bone loss by promoting osteogenic differentiation. BioMed Pharmacother (2022) 146:112524. doi: 10.1016/j.biopha.2021.112524 34906775

[138] Guo A Li K Xiao Q. Fibroblast growth factor 19 alleviates palmitic acid-induced mitochondrial dysfunction and oxidative stress *Via* the Ampk/Pgc-1alpha pathway in skeletal muscle. Biochem Biophys Res Commun (2020) 526(4):1069–76. doi: 10.1016/j.bbrc.2020.04.002 32305136

